# Positive predictive value of mammographic features on digital breast tomosynthesis

**DOI:** 10.1186/bcr3778

**Published:** 2015-11-05

**Authors:** Bhavna Batohi, Juliet Morel, Reema Wasan, Asif Iqbal, David Evans, Jane Goligher, Michael Michell, Clare Peacock, Rumana Rahim, Keshthra Satchithananda

**Affiliations:** 1 King's College Hospital, London, UK

## Introduction

Digital breast tomosynthesis (DBT) is increasingly used for the further assessment of mammographically detected abnormalities due to its superior specificity compared with 2D digital mammography (2DDM). In this study we evaluate the positive predictive value (PPV) of mammographic features on DBT and assessment categories as per the Royal College of Radiologists (RCR) breast group classification system.

## Methods

Women recalled following routine screening mammograms underwent bilateral 2DDM and DBT over an 18-month period. Experienced screening radiologists prospectively evaluated each case, documenting mammographic sign, size and classification according to the RCR breast group guidelines. DBT findings and final pathology were then correlated.

## Results

A total of 759 abnormalities were included. On DBT, 221 (29 %) were normal. Of the remaining 538, there were 207 circumscribed masses, 89 spiculate masses, 156 microcalcifications, 35 distortions and 51 asymmetric densities. Final histology revealed 204 malignant and 334 benign lesions. The PPVs were 97.7 % for spiculate masses, 65.7 % for distortions, 35.8 % for microcalcifications, 16.9 % for circumscribed masses and 5.8 % for asymmetric densities.

## Conclusion

DBT allows more accurate assessment of mammographic lesions without the impedance of overlying tissues. Spiculate masses have the highest PPV on both 2DDM and DBT. Although the PPV for asymmetric densities appears relatively low on DBT, this is still nearly twice that of 2DDM. Better understanding of the likelihood of tomographic signs indicating malignancy will increase the value of DBT.

